# Development and evaluation of an automated phenylephrine delivery system by lower limit control for managing intraoperative hypotension

**DOI:** 10.1007/s00540-025-03476-z

**Published:** 2025-03-12

**Authors:** Osamu Nagata, Emi Morinushi, Aya Kuroyanagi, Fumiyo Yasuma

**Affiliations:** 1 Department of Anesthesia, Touto Kasukabe Hospital, 652-7 Ohata, Kasukabe, Saitama 344-0022 Japan; 2 Department of Anesthesiology and Pain Relief Center, The University of Tokyo Hospital, 7-3-1 Hongo, Bunkyo-ku, Tokyo 113-8655 Japan; 3 Department of Anesthesiology, Saitama Cooperative Hospital, 1317 Kizoro, Kawaguchi, Saitama 333-0831 Japan; 4 Department of Anesthesiology, Center Hospital of the National Center for Global Health and Medicine, 1-21-1 Toyama, Shinjuku-ku, Tokyo 162-8655 Japan

**Keywords:** Automated drug delivery system, Phenylephrine, Intraoperative hypotension, Noninvasive blood pressure measurement, Lower limit control

## Abstract

**Purpose:**

In this study, we aimed to develop and evaluate an automated phenylephrine delivery system by lower limit control for the management of intraoperative hypotension, assessing its efficacy in maintaining adequate blood pressure levels.

**Methods:**

Twenty patients undergoing surgery with anticipated blood pressure fluctuations were enrolled in this study. Patients were randomly assigned to two groups. Noninvasive blood pressure (NIBP) was measured at 2.5-min intervals using an upper arm cuff. In the automated group, phenylephrine administration was governed by an automated system that delivered bolus doses and adjusted the continuous infusion rate when mean blood pressure (MBP) dropped below 65 mmHg. In the manual group, phenylephrine administration was initiated by the attending anesthesiologist under the same MBP threshold. Propofol, remifentanil, and rocuronium were administered via the automated delivery system for total intravenous anesthesia, to minimize hemodynamic variability between groups. The primary end point was the percentage of time during which MBP remained above 65 mmHg and systolic blood pressure below 140 mmHg, measured from the initiation to the cessation of intravenous anesthesia and assessed using a non-inferiority test.

**Results:**

The automated group adequately maintained blood pressure within the target range for 84.53% of the time, compared to 72.45% in the manual group, confirming statistical non-inferiority (*p* < 0.001).

**Conclusion:**

This system effectively managed intraoperative hypotension using intermittent NIBP measurements, which are more feasible in clinical practice. Despite relying on less frequent and lower-resolution blood pressure data, it demonstrated efficacy comparable to anesthesiologist-led management, indicating its potential for broader clinical application.

## Introduction

Intraoperative hypotension (IOH) is a prevalent perioperative event that significantly contributes to postoperative morbidity. Current guidelines advocate maintaining a mean arterial pressure (MAP) of ≥65 mmHg to mitigate the risk of adverse outcomes [1]. Anesthesiologists typically manage IOH by administering vasoactive agents, such as phenylephrine, via peripheral intravenous access to rapidly restore arterial pressure [2] while simultaneously identifying the underlying etiology of hypotension to guide further therapeutic interventions [2]. The minimum necessary dose of vasopressors is administered repeatedly and continuously, combined with multiple methods to prevent abnormal hypotension [2]. However, when anesthesiologists are occupied with procedures such as tracheal intubation, they may be unable to administer a vasopressor immediately, even if the automatically measured blood pressure is decreasing, leading to delays in controlling hypotension.

To prevent such delays and enhance the accuracy of blood pressure control, attempts to automate blood pressure management using specialized equipment have been reported. In a study of patients undergoing cesarean section managed with spinal anesthesia [3], noninvasive blood pressure (NIBP) was measured at 1-min intervals during a markedly limited evaluation period from the time of spinal injection to the time of uterine incision, and the systolic blood pressure (SBP) was successfully maintained within the range (baseline±20%) in 94.6% of the observation period by administering continuous phenylephrine at 0.1 mg/min through on–off control. In another study [4], a system that controls the rate of continuous administration of vasopressors and the rate of infusion fluid administration every few seconds was developed using proportional-integral-derivative (PID) control to ensure that the mean blood pressure (MBP) value following general anesthesia matched the target value, by setting the target MBP value before surgery. In these studies, a target blood pressure level was assigned to each patient, which poses the risk of administering an excessively low target to patients with initially low blood pressure, and an excessively high target to those with initially high blood pressure, potentially resulting in the maintenance of unnecessarily elevated blood pressure. These systems control the administration of vasopressors every few seconds or tens of seconds based on measured arterial blood pressure values. However, in routine clinical practice, blood pressure is typically measured intermittently using a cuff every 2–5 min, and continuous evaluation via invasive measurement is limited to certain cases. In the management of IOH, a lower limit control algorithm that maintains the MBP above a threshold to prevent an increased risk of postoperative complications is appropriate. Moreover, adjusting the vasopressor administration rate to a higher frequency than normal to precisely align the measured blood pressure with a predetermined target value is unnecessary. Therefore, a system that controls vasopressor administration based on NIBP measurements taken at few minute intervals, as commonly practiced, is clinically advantageous because it can be applied to a broader patient population.

Thus, we developed a system that automatically regulates the repeated and continuous administration of phenylephrine via a peripheral vein, aiming to promptly restore blood pressure when hypotension is detected with a cuff using NIBP measurements. Furthermore, we conducted a non-inferiority randomized controlled trial to evaluate the efficacy and safety of this system compared to anesthesiologist-managed blood pressure control, focusing on the percentage of time abnormal hypotension was detected.

## Methods

### Research protocol

This study was approved by the Certified Review Board (CRB3200011) of the National Center for Global Health and Medicine and registered as a specific clinical study (jRCTs032220082) in the Japan Registry of Clinical Trials. We included patients aged ≥20 years with American Society of Anesthesiologists’ physical status (PS) I–III; without significant cardiovascular comorbidities; scheduled for elective surgery under total intravenous anesthesia (TIVA) with propofol, remifentanil, and rocuronium; and who did not meet the exclusion criteria outlined in Table 1. Informed consent was obtained from all participants before assigning them via randomization into either the manual or automated control group. The study employed a single-center, single-blinded, randomized, parallel-group design, with only the patients blinded to group allocation.

**Table 1:** Exclusion criteria

1) History of hypersensitivity to propofol, remifentanil, rocuronium, sugammadex, vasoactive agents (e.g., phenylephrine, noradrenaline), or intravenous infusion products
2) Inability to apply a bispectral index sensor intraoperatively
3) Patients unable to receive additional doses of rocuronium following the administration
4) Inability to perform intraoperative blood pressure monitoring
5) Patients scheduled for hypothermic surgery
6) Patients scheduled for cardiovascular surgery
7) Pregnant or lactating patients
8) Patients with significantly impaired cardiac function (ejection fraction < 40%), a history of ventricular tachycardia, or coronary intervention within the past 3 months
9) Any patients deemed inappropriate for inclusion by the investigator

### Clinical application of the system

**Fig. 1:**
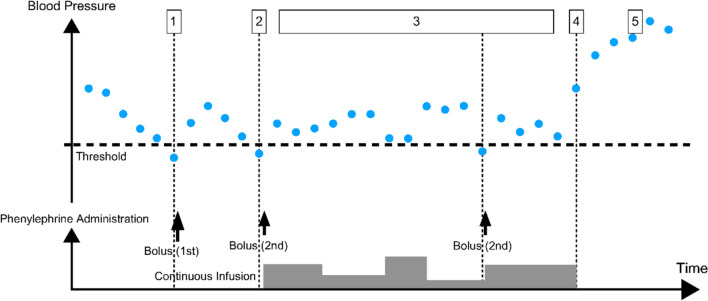
Conceptual framework for automated phenylephrine administration. 1. Upon detection of mean blood pressure (MBP) falling below the predefined threshold, the system administers the first bolus of phenylephrine. 2. If MBP remains below the threshold, a second phenylephrine bolus is administered, followed by the initiation of continuous phenylephrine infusion. 3. The phenylephrine infusion rate is adjusted based on subsequent MBP readings. 4. Continuous phenylephrine administration is discontinued when MBP is sufficiently above the threshold. 5. The device remains inactive if MBP exceeds the upper safety limit

The system developed in this study is a lower limit control system that administers phenylephrine when the measured mean blood pressure falls below a threshold value (Fig. 1). It does not set a target blood pressure value for each patient or administer vasopressors to maintain constant blood pressure as in previous reports [3, 4]. We used a model-free control as it is difficult to create a mathematical model to explain blood pressure fluctuations in patients in a clinical setting; even if one were created, the accuracy of the model could not be guaranteed. Since the objective of this system is lower limit control, a rule-based, closed-loop, data-driven control algorithm was implemented instead of classical methods, such as PID control. As this represents the first stage of development, the system lacks the capability to predict a drop in blood pressure several minutes after multiple measurements and to administer vasopressors prophylactically. Notably, the system was designed to assist anesthesiologists and other clinicians in hemodynamic management and was not intended to diagnose or treat the underlying cause of hypotension.

**Fig. 2:**
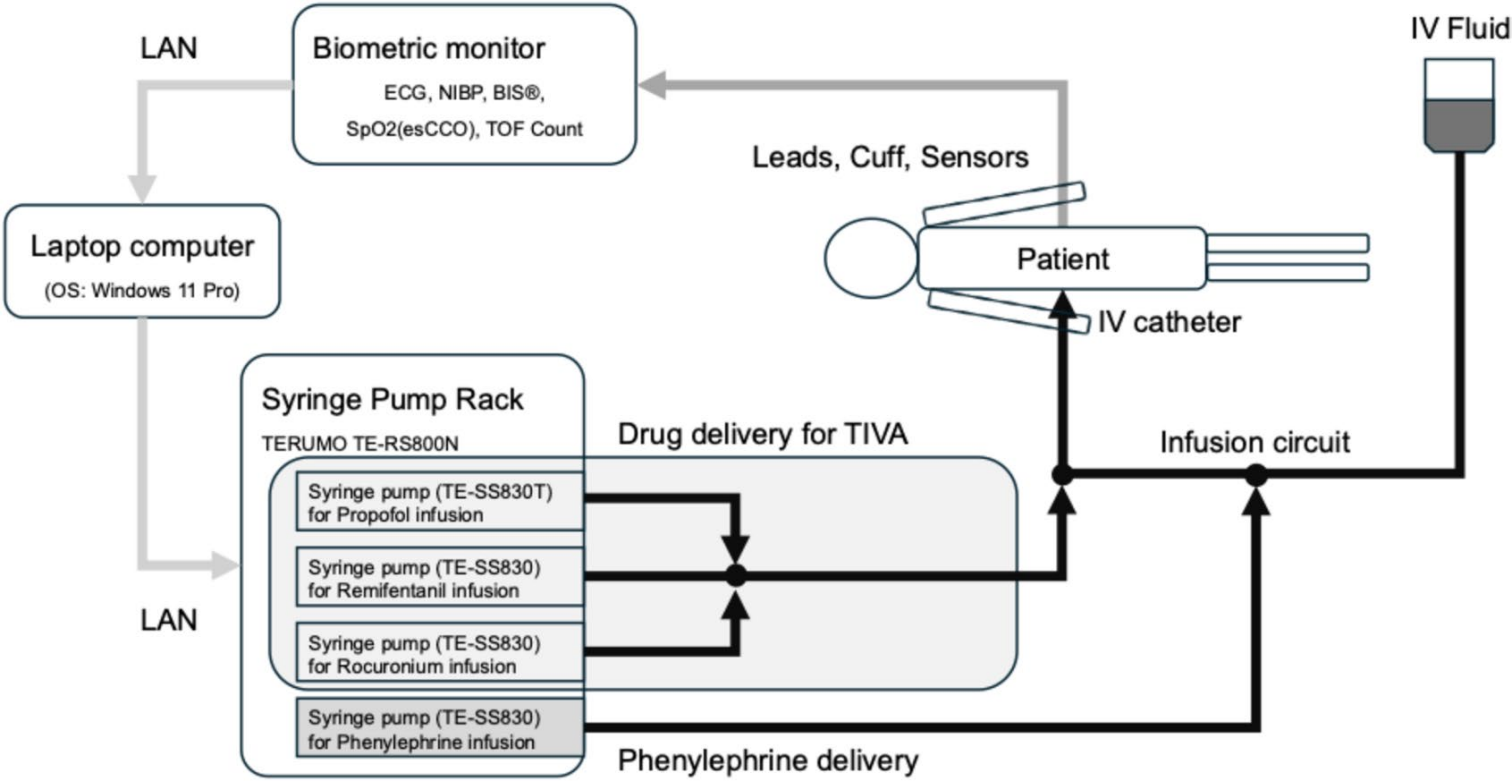
The configuration of this system. *LAN* local area network, *TIVA* total intravenous anesthesia

In this system (Fig. 2), information from the biometric monitor (BSM-6701/CSM-1701, Nihon Kohden Corporation, Tokyo, Japan) is provided via LAN to the control software running on a laptop computer with Windows 11 Professional, as in the anesthesia recording system. The control software calculates the phenylephrine dose using programmed rule-based calculations each time NIBP is measured at 2.5-min intervals. If the blood pressure cannot be measured due to a measurement error, the control state is not changed. If an abnormal value is measured, the dosage rate is calculated accordingly, and the dosage rate is also corrected at the next measurement, or a temporary re-measurement by the anesthesiologist. The calculation results are transferred to the syringe pump (TE-SS830, Terumo Corporation, Tokyo, Japan) through a communication rack (TE-RS800N, Terumo Corporation, Tokyo, Japan) via LAN, and the administration rate of phenylephrine solution (0.05 mg/mL) is changed. This control software was added to the automated delivery system for TIVA [5], which changes the dosing rate of three syringe pumps filled with propofol, remifentanil, and rocuronium solutions.

### Intraoperative patient management

We utilized an automated delivery system for TIVA [5] to objectively standardize the administration of general anesthetics, including propofol, remifentanil, and rocuronium. We incorporated a function to automatically manage the dosage of vasopressors based on our original algorithm for this system and were able to eliminate the effects of the anesthesiologist's arbitrary decisions on the administration of anesthetics.

Upon admission to the operating room, an intravenous infusion circuit is connected to administer intravenous anesthetics and vasoactive drugs by securing the intravenous line (≥20G in diameter). Using an infusion pump (TE-LM835A, Terumo Corporation, Tokyo, Japan), 1% glucose-acetate Ringer's solution is administered at an initial rate of 10 mg/kg/h from the infusion circuit. The patient received 6 L/min of oxygen through a face mask, and a cuff for NIBP measurement was applied to the upper arm. Electrodes for muscle relaxation monitoring (NM-345Y, Nihon Kohden Corporation, Tokyo, Japan) were placed on the forearm and hand. An electrode for electroencephalogram measurement (BIS Quattro Sensor, Covidien Japan Corporation, Tokyo, Japan) was attached to the forehead to monitor bispectral index (BIS) values. An SpO_2_ adhesive sensor (TL-281T-1B, Nihon Kohden Co., Ltd., Tokyo, Japan) was placed on the fingertips for calibration, and the noninvasive estimated continuous cardiac output (esCCO) system [6] was used to measure circulatory parameters including estimated continuous cardiac index (esCCI) and estimated stroke volume index (esSVI). After adequate oxygenation and denitrogenation, continuous remifentanil administration was initiated using the automated delivery system for TIVA [5], followed by propofol administration, also controlled by the system. Following calibration of the muscle relaxation monitor after the patient was anesthetized, muscle relaxation with rocuronium was achieved, and tracheal intubation was performed. Depending on the progress of the surgery and the state of the anesthesia, the anesthesiologist changed the infusion rate of 1% glucose-containing acetic acid Ringer’s solution based on empirical rules while referring to fluctuations in blood pressure. In addition, antibiotics, single-dose drugs, and additional infusion products were administered through the side tubes of the infusion route as needed. Subsequent administration of propofol, remifentanil, and rocuronium was regulated by the automated delivery system for TIVA through closed-loop control, utilizing BIS values and train-of-four count as indicators [5].

### Treatment of abnormal hypotension

In this study, NIBP was measured every 2.5 min in all patients. MBP <65 mmHg was defined as abnormal hypotension, while SBP >140 mmHg was classified as hypertension.

In the manual group, when MBP indicated abnormal hypotension, phenylephrine was administered as a single injection of up to 0.1 mg or as a continuous infusion (maximum 2 mg/h) at the discretion of the anesthesiologist with board certification. Phenylephrine was initiated only after the onset of abnormal hypotension, and prophylactic administration in the absence of hypotension was prohibited.

In the automated group, the device automatically regulated phenylephrine administration according to the following algorithm (Fig. 1):

- When MBP is abnormally low, the control device enters vasopressor administration mode, administering an initial dose of 0.002 mg/kg of phenylephrine (maximum 0.1 mg). If hypotension persists, an additional dose of half the initial amount is administered, followed by a continuous infusion at a rate determined by the protocol.
- If the anesthesiologist determined that automated administration was insufficient to raise the pressure, a manual dose of phenylephrine was administered and recorded along with the time.
- If MBP recovers from hypotension, continuous phenylephrine administration is maintained or resumed at the protocol-determined dosing rate if MBP is < 85 mmHg. If MBP exceeds 85 mmHg, continuous administration is suspended, following protocol calculations.
- If continuous phenylephrine administration is interrupted for > 15 min, the system exits vasopressor administration mode and returns to normal.

If the anesthesiologist judged that the patient could not recover from hypotension with continuous phenylephrine administration, the initiation of continuous noradrenaline administration was considered.

### Statistical analysis

The evaluation period for statistical analysis spanned from the initiation of remifentanil to the completion of propofol/remifentanil administration. The full analysis set (FAS), which represents the maximum analysis population, included patients who did not receive phenylephrine and experienced no episodes of abnormal hypotension during the evaluation period. These patients were considered to be in an unusually favorable regulatory situation, with an abnormal hypotension time rate of 0, despite no interventions being made to avoid hypotension. Consequently, these patients were excluded from the protocol per set (PPS). Patients who required continuous noradrenaline administration were also excluded from PPS. Missing NIBP values were left as missing and not included in the evaluation period.

The primary end point, the adequate blood pressure time percentage, was defined as the percentage of time during the evaluation period when MBP >65 mmHg and SBP <140 mmHg were maintained. This value was calculated by subtracting the percentage of time during the evaluation period when either abnormal hypotension (MBP <65 mmHg) or hypertension (SBP >140 mmHg) occurred. The non-inferiority of the automated group compared to that of the manual group was assessed using a t test with a non-inferiority margin of 10% and a significance level of 5%.

Secondary end points included the percentage of cases requiring vasopressors other than phenylephrine, use of additional vasopressors administered by the anesthesiologist, total dose of phenylephrine and infusions, percentage of time with adequate anesthesia from the start to the end of surgery, esCCI, esSVI, and MBP variability.

For safety evaluation, adverse events occurring from the day of surgery until 48 h postoperatively were assessed. Adverse events were evaluated based on the date of onset, event name, severity, treatment, outcome, and the causal relationship to the study. They were categorized as adverse events observed in subjects, adverse events observed in non-subjects, and study equipment malfunctions.

### Sample size design

Due to the absence of previous or similar studies, we assumed that the regulatory performance would be comparable between the manual and automated groups, as described in a developmental study of the automated delivery system for TIVA [7]. We calculated a power of 90%, a non-inferiority margin of 10%, and a dropout rate of 20% (e.g., no circulatory agonist administered). The required sample size was calculated to be 20 patients in total.

## Results

**Fig. 3:**
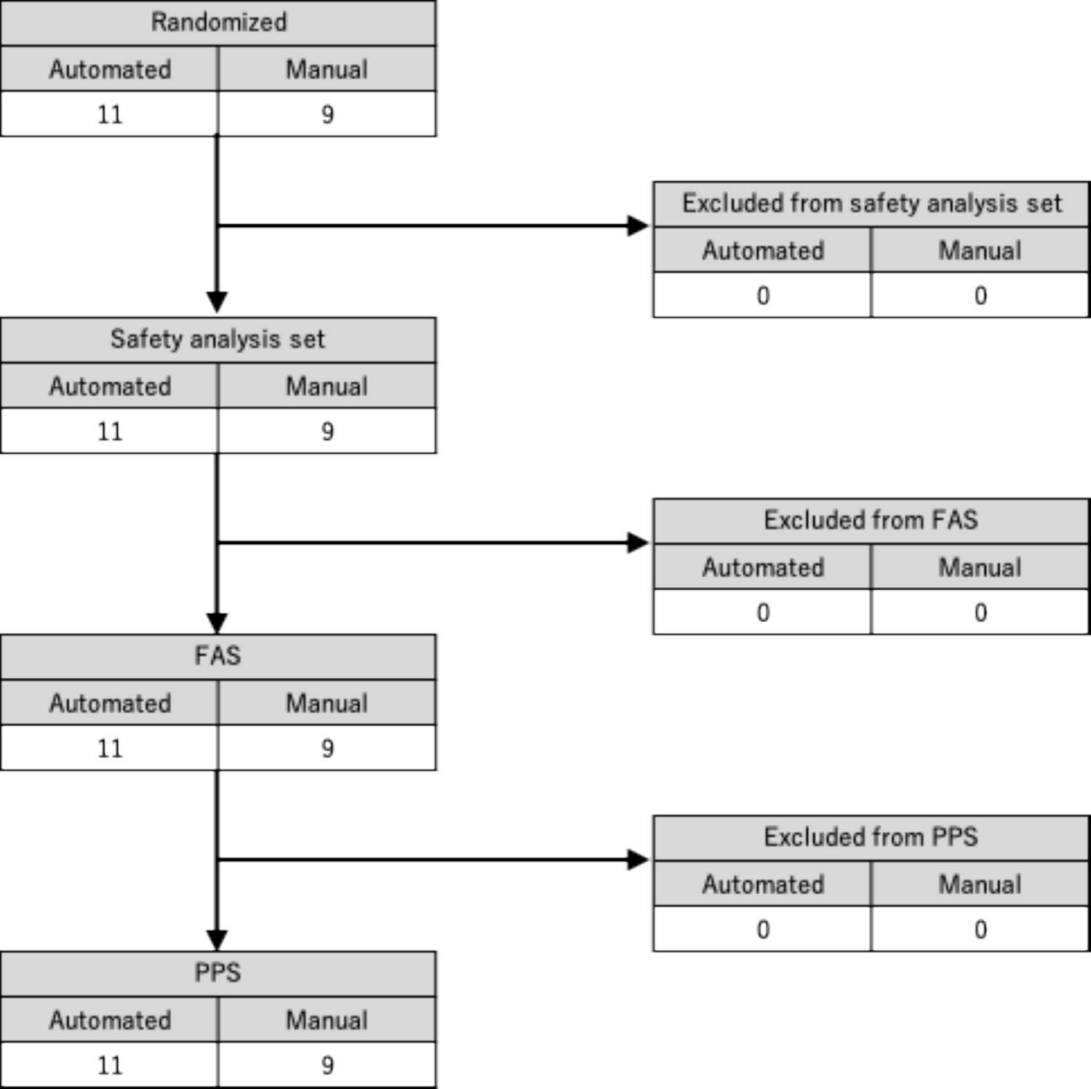
Patient flowchart. *FAS* full analysis set, *PPS* per protocol set

Twenty patients were enrolled in this study (Fig. 3). Since no cases were excluded from the safety analysis population, FAS, or PPS, the analysis population aligned with the FAS, PPS, and safety analysis populations.

### Patient characteristics

The characteristics of the patients are shown in Table 2. In terms of surgical procedures, four and three patients in the manual and automated groups, respectively, underwent hip replacement, which significantly impacts the circulatory dynamics. Additionally, two patients in the manual group and one in the automated group underwent surgery using a tourniquet. One and two patients in the automated and manual groups, respectively, underwent laparoscopic surgery in the head-up position, while three patients in the automated group underwent the same in the head-down position. One patient in the manual group and three in the automated group underwent other procedures.

**Table 2:** Patient demographics and surgical characteristics

Background factors		Manual group (*n* = 9)	Automated group (*n* = 11)	*p* value
Sex	Male	3 (33.3%)	6 (54.5%)	0.406
	Female	6 (66.7%)	5 (45.5%)	
Age (year old)	Mean ± SD	68.1 ± 8.4	71.1 ± 7.1	0.402
	[Min–max]	[49–79]	[56–83]	
Height (cm)	Mean ± SD	158.2 ± 11.4	162.4 ± 10.7	0.404
	[Min–max]	[145.5–174.5]	[147.5–180.0]	
Body weight (kg)	mean ± SD	63.4 ± 13.8	66.6 ± 9.7	0.556
	[Min–max]	[43.1–84.0]	[49.0–87.0]	
BMI (kg/m^2^)	Mean ± SD	25.5 ± 5.9	25.3 ± 3.2	0.913
	[Min–max]	[17.1–35.5]	[21.9–30.8]	
ASA physical status (PS)	1	0 (0.0%)	1 (9.1%)	0.230
	2	7 (77.8%)	8 (72.7%)	
	3	2 (22.2%)	2 (18.2%)	
Duration of evaluation (min)	Mean ± SD	165.5 ± 87.3	188.4 ± 106.9	0.612
	[Min–max]	[86.2–359.6]	[92–415.5]	

*SD* standard deviation

### Changes in blood pressure during general anesthesia

**Fig. 4:**
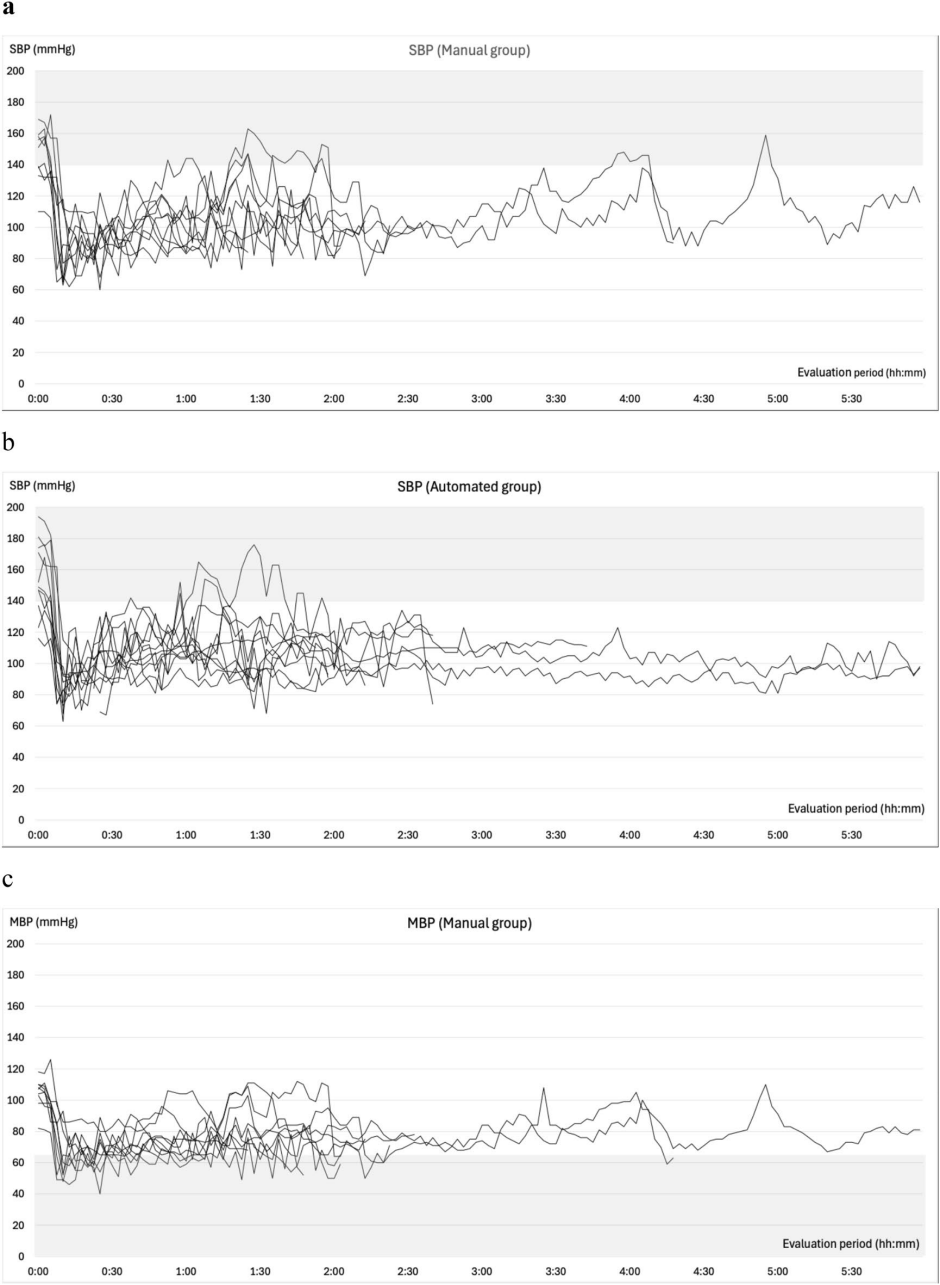

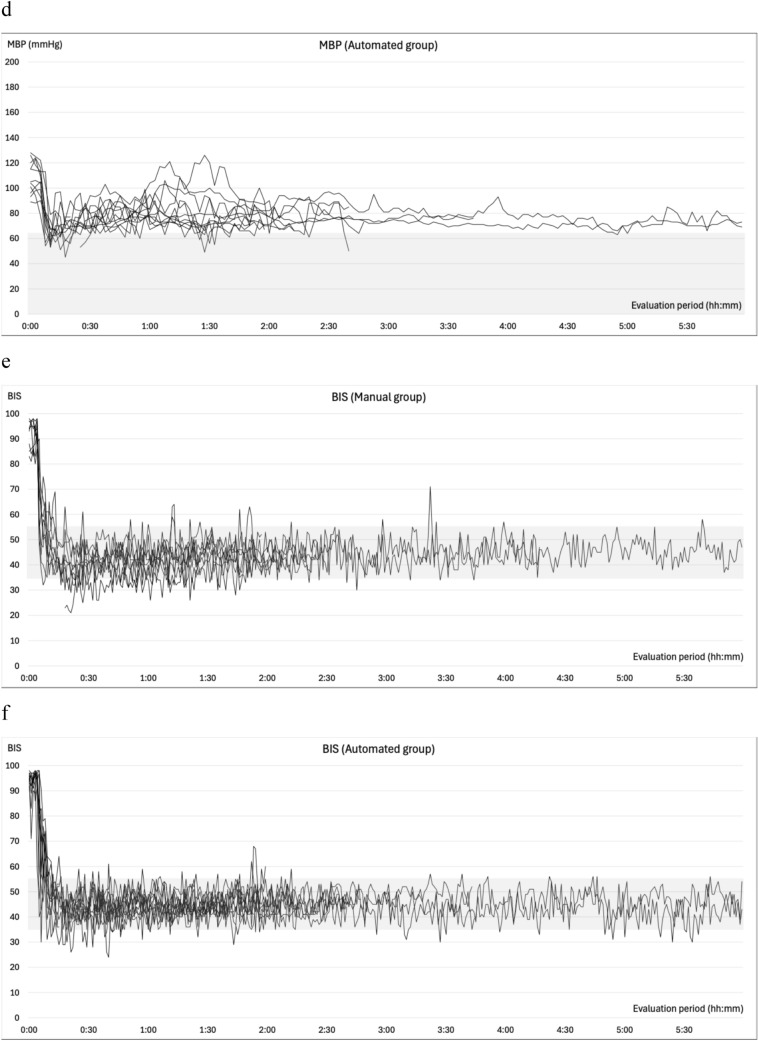

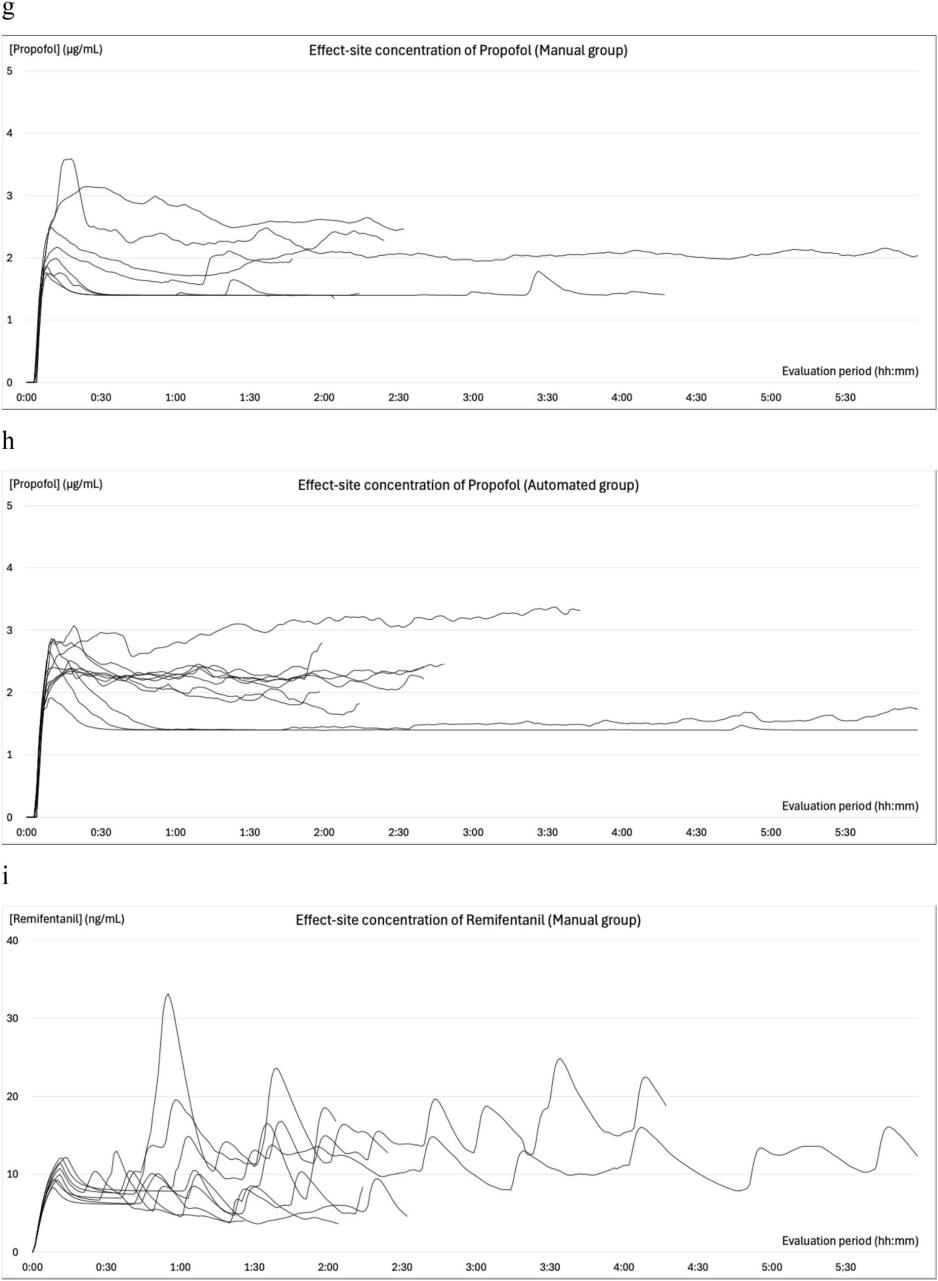

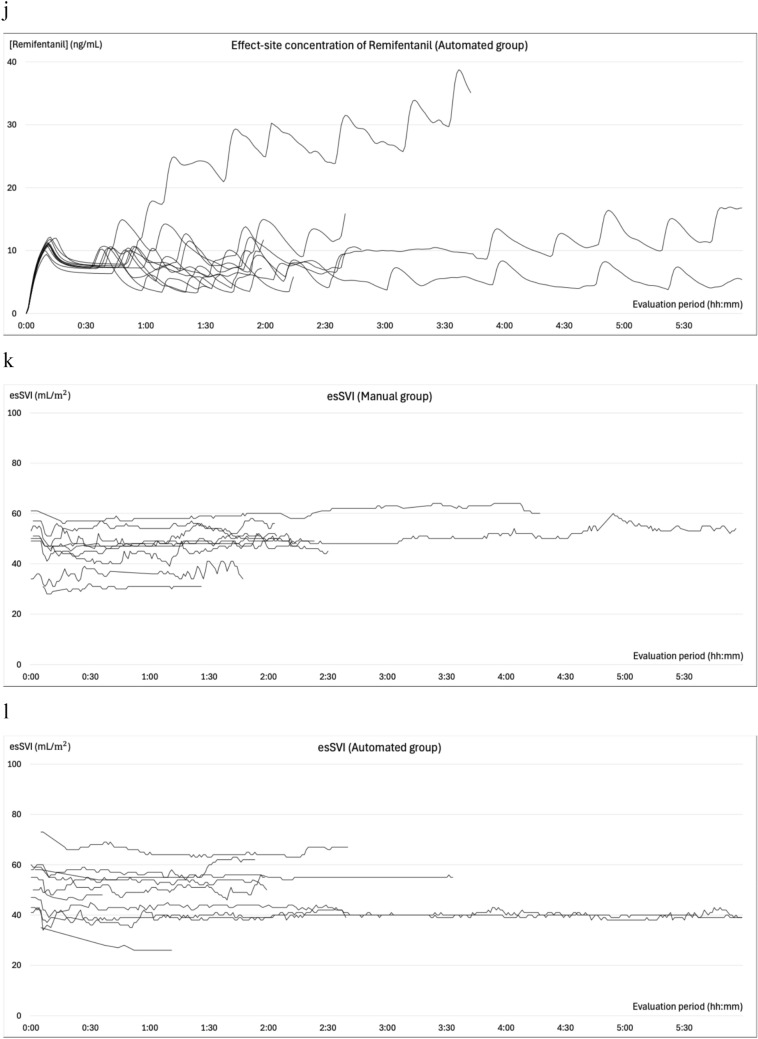

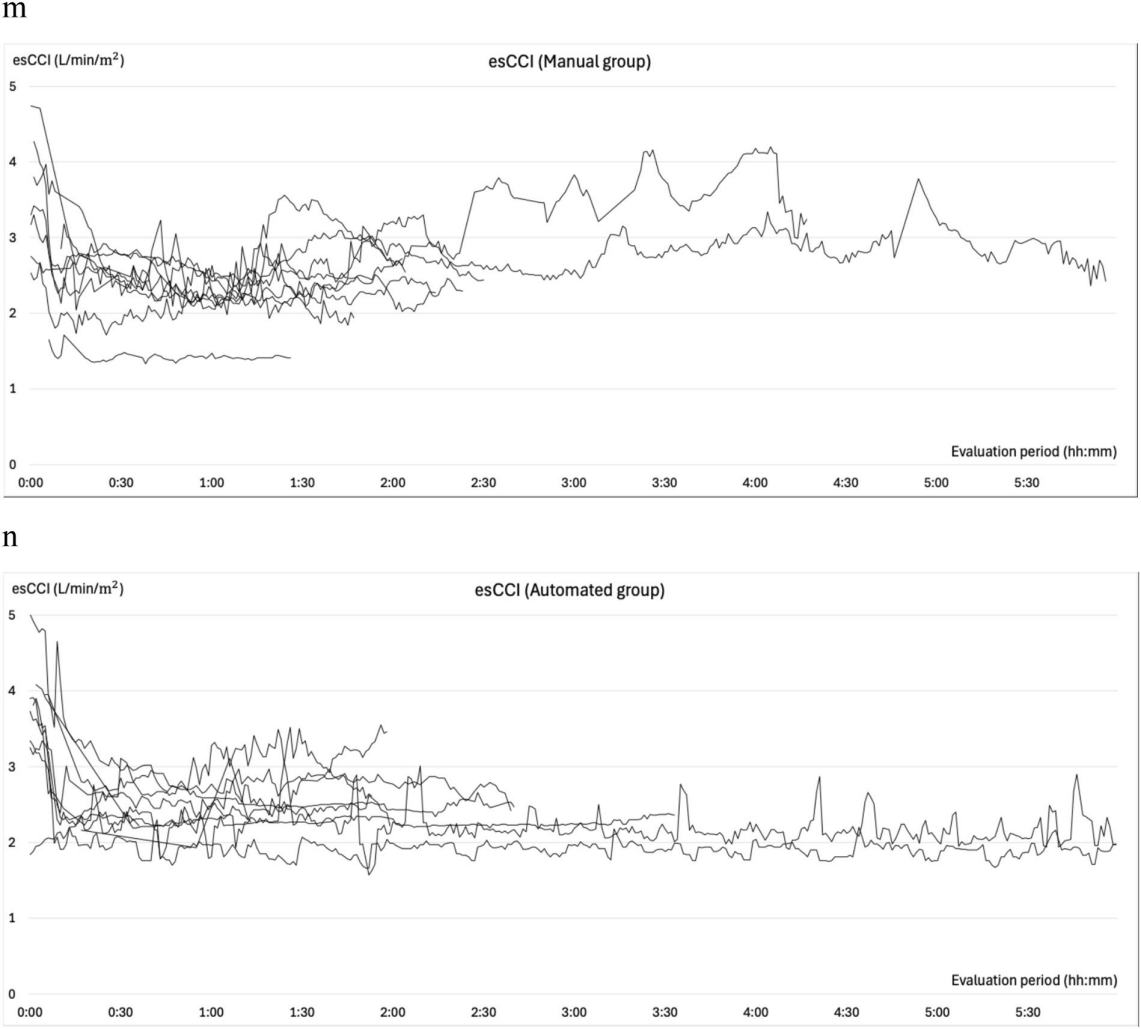
Intraoperative trends. Panels **a** and **b** depict the changes in systolic blood pressure, while panels **c** and **d** illustrate the variations in mean blood pressure from the initiation to the conclusion of the evaluation period. Panels **e** and **f** illustrate the variations in BIS®, **g** and **h** show the change of effect site concentration of propofol, **i** and **j** reveal the change of effect site concentration of remifentanil under automated delivery system for TIVA. Panels **k** and **l** depict the changes in the stroke volume index (esSVI), and **m** and **n** in the cardiac index (esCCI). Data are presented for the manual (**a**, **c**, **e**, **g**, **i**, **k**, **m**) and automated (**b**, **d**, **f**, **h**, **j**, **l**, **n**) groups, with each line representing individual patient data. SBP: systolic blood pressure; MBP: mean blood pressure

The changes in SBP, MBP, BIS, effect site concentrations of propofol and remifentanil, esSVI, and esCCI are illustrated in Fig. 4. All patients experienced at least one episode of abnormal hypotension. In the manual group, 99 measurements were categorized as abnormal hypotension: 29 (55%) were single events, 13 (24%) two consecutive events, 3 (6%) three consecutive events, and 8 (15%) ≥consecutive events. Conversely, in the automated group, 52 cases of abnormal hypotension were recorded, of which 34 (80%) were single events, 4 (10%) two consecutive events, and 4 (10%) three consecutive events. Hypertension was observed in six (66.7%) patients in the manual group and eight (72.7%) in the automated group prior to anesthesia induction. The occurrences of intraoperative hypertension were recorded as 12 (four patients) in the manual group and 8 (four patients) in the automated group. Hypertension mostly occurred during subcutaneous administration of adrenaline-containing local anesthetics, during positional changes, or during blood flow occlusion with cuffs. Hypertension related to phenylephrine administration during episodes of abnormal hypotension was recorded once (one patient) in the manual group and thrice (two patients) in the automated group, all with an MBP of <100 mmHg. In the automated group, only a single (one patient) instance of hypertension within 10 min following a single dose of phenylephrine was recorded, and two (one patient) were observed during continuous phenylephrine administration.

### Primary end point

**Fig. 5:**
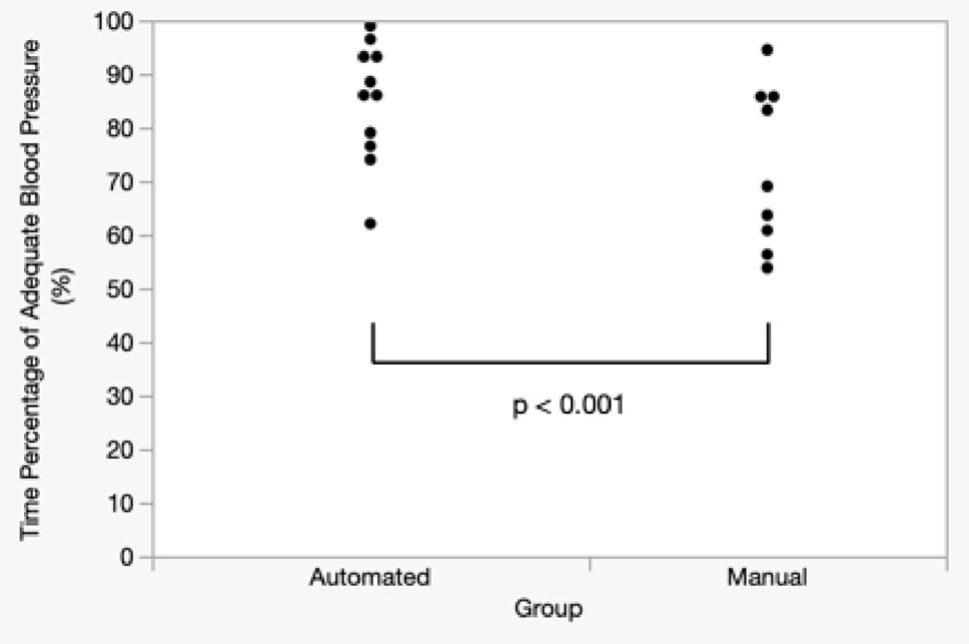
Primary outcome: distribution of time spent (in percentages) within the target blood pressure range

The distribution of the adequate blood pressure time percentage is presented in Figure 5. The mean and standard deviation (SD) for the automated and manual groups were 84.53% (11.05) and 72.45% (14.93), respectively, with a 97.5% lower limit of the one-sided confidence interval of -0.12%. The automated group was deemed non-inferior to the manual group (*p* <0.001).

### Secondary end point

**Fig. 6:**
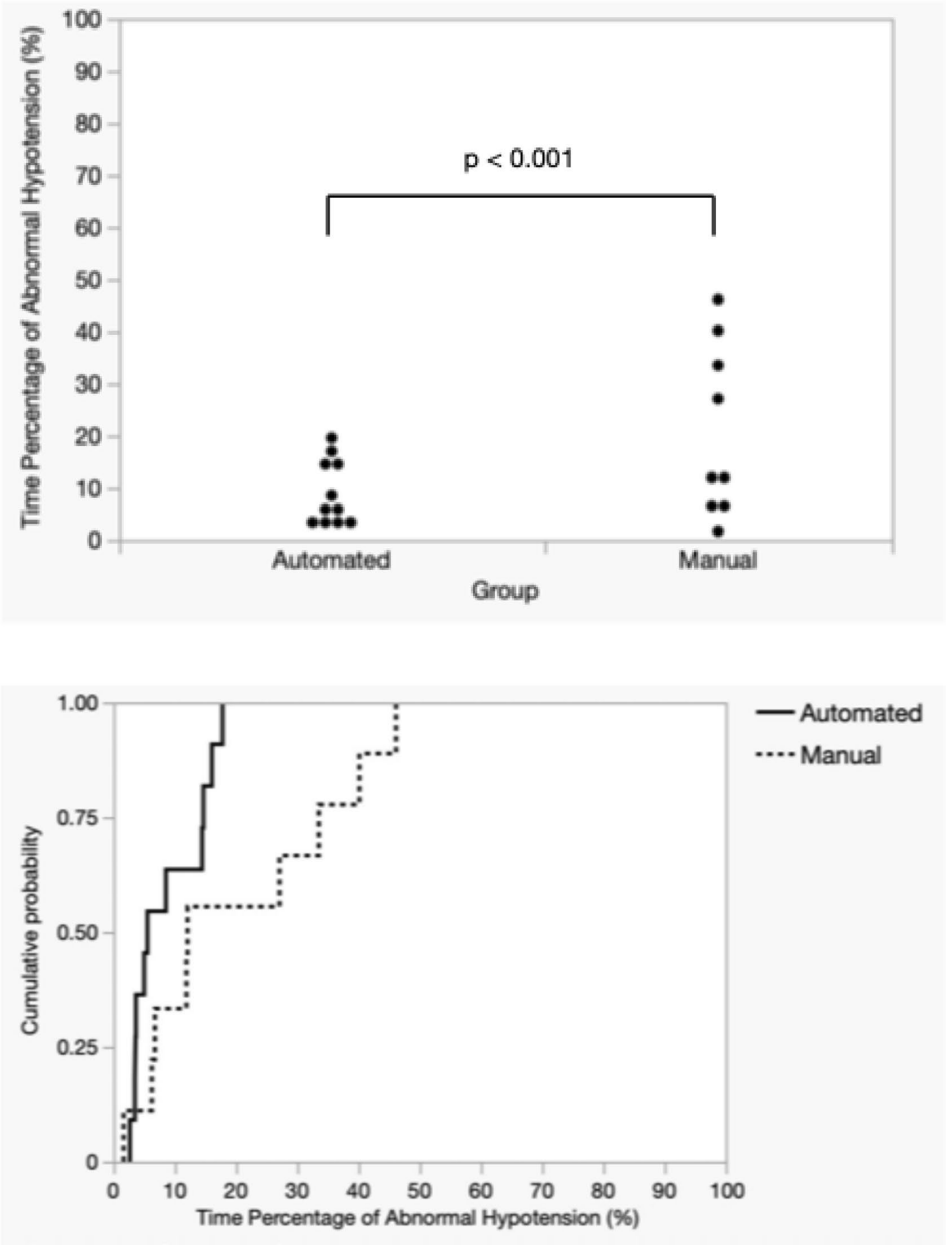
Distribution and cumulative probability of time spent (in percentages) in hypotension

**Fig. 7:**
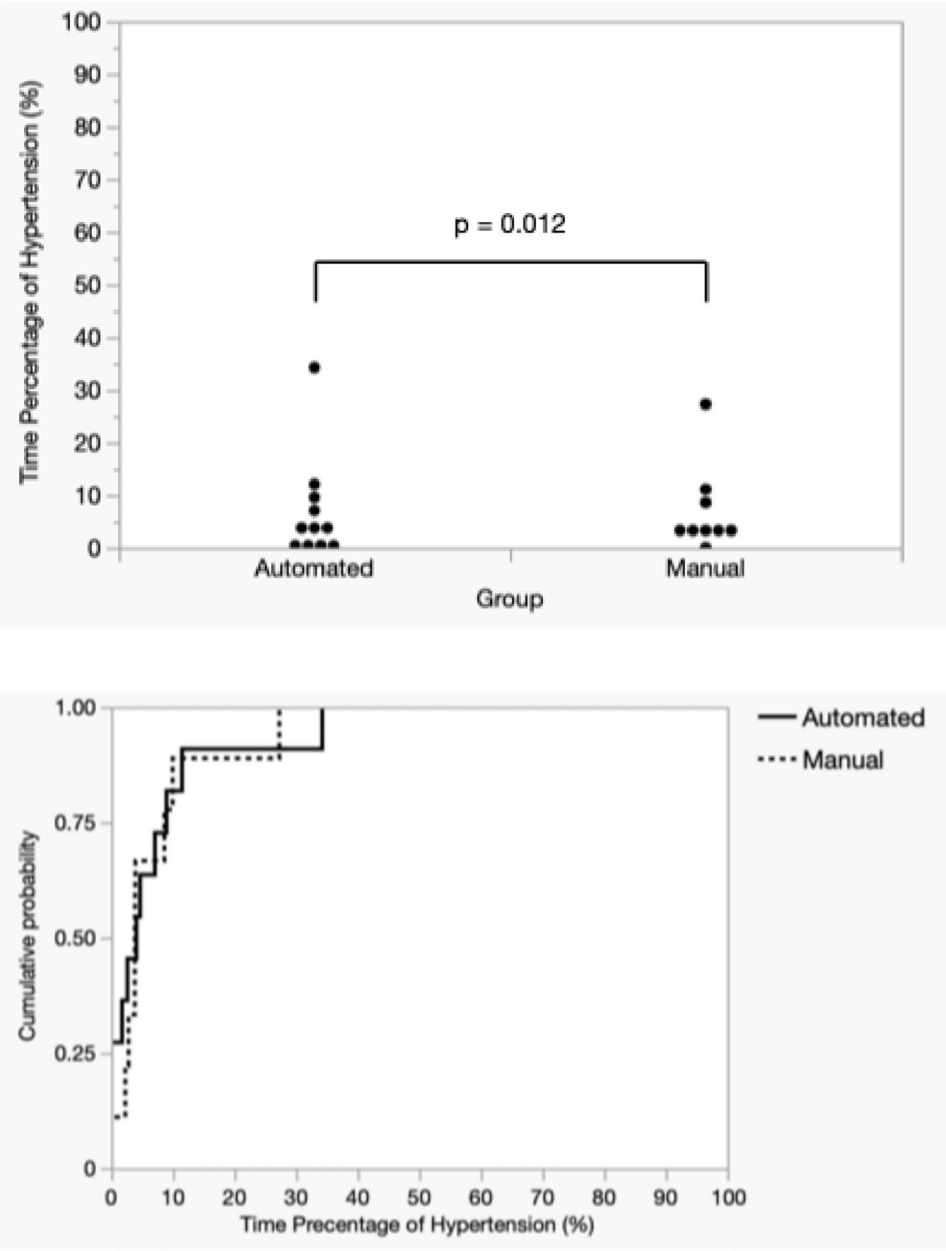
Distribution and cumulative probability of time spent (in percentages) in hypertension

Figures 6 and 7 display the distribution and cumulative probability plots of the abnormal hypotension and hypertension time percentages for both groups, calculated for each participant. For the abnormal hypotension time percentage, a significant discrepancy was observed between the cumulative probability plots of the manual and automated groups, with the mean (SD) of 20.62% (16.39) for the manual group being more than double that of the mean 8.67% (5.87) for the automated group. Contrarily, the mean values for the percentage of hypertensive time in the manual and automated groups were 6.93% (8.22) and 6.80% (9.86), respectively, with cumulative probability plots overlapping, indicating a comparable distribution. The automated group was judged to be non-inferior to the manual group regarding the abnormal hypotension time percentage and the hypertension time percentage (abnormal hypotension: *p* < 0.001, hypertension: *p *= 0.012). The 95% confidence interval for the difference in the abnormal hypotension time percentage ranged from -23.06 to -0.83 and did not include 0, indicating a significant difference in outcomes.

The automated group (87.35 ± 10.59) was found to be non-inferior to the manual group (78.73 ± 17.72) with regard to the percentage of time that adequate anesthetic conditions were maintained from the start to the end of surgery (*p*=0.005). No patient in either group received vasoactive drugs (such as noradrenaline) other than phenylephrine. No additional phenylephrine was administered by the anesthesiologist during the evaluation period in the automated group. There was no significant difference between the two groups in hourly doses of phenylephrine and infusions, esSVI, esCCI, or MBP (Table 3).

**Table 3:** Additional outcome variables (including phenylephrine administration, infusion therapy parameters, circulatory parameters, and mean blood pressure during the evaluation period)

	Manual group (*n* = 9)	Automated group (*n* = 11)	*p* value
Propofol dose (mg/kg/h)	4.43 ± 1.19	4.86 ± 1.11	*p* = 0.417
Mean ± SD, [min–max]	[3.06–6.15]	[2.87–6.63]	
Remifentanil dose (µg/kg/min)	0.32 ± 0.12	0.28 ± 0.11	*p* = 0.476
Mean ± SD, [min–max]	[0.17–0.50]	[0.18–0.57]	
Total phenylephrine dose (mg)	0.73 ± 0.63	2.52 ± 3.17	*p* = 0.113
mean ± SD, [min–max]	[0.10–2.0]	[0.27–10.75]	
Phenylephrine dose (mg/h)	0.35 ± 0.37	0.76 ± 0.58	*p* = 0.085
mean ± SD, [min–max]	[0.02–0.98]	[0.10–1.62]	
Total infusion volume (mL)	1361 ± 599	1787 ± 1396	*p* = 0.406
mean ± SD, [min–max]	[700–2600]	[750–5370]	
Infusion volume (mL/h)	517 ± 126	556 ± 178	*p* = 0.589
mean ± SD, [min–max]	[391–800]	[281–880]	
Cardiac Index (esCCI) (L/min/m^2^)	2.44 ± 0.47	2.50 ± 0.36	*p* = 0.736
mean ± SD, [min–max]	[1.42–3.05]	[1.94–3.25]	
Stroke Volume Index (esSVI) (mL/m^2^)	46.8 ± 8.9	48.2 ± 10.7	*p* = 0.769
mean ± SD, [min–max]	[30.5–60.0]	[28.4–65.2]	
Mean blood pressure (MBP) (mmHg)	74.9	78.5	*p* = 0.227
mean ± SD, [min–max]	[65.2–87.9]	[72.0–92.3]	

*SD* standard deviation

### Safety evaluation

No serious adverse event or adverse event causally related to the study device were reported in this study. Hypotension during general anesthesia was not classified as an adverse event, as it was a predefined end point of this study. The most common adverse events included abnormal postoperative blood test results (18 patients, 90%), pain (17 patients, 85%), and nausea and vomiting (five patients, 25%). Study-related adverse events included an intraoperative injection device malfunction in one patient in the manual group, which did not have serious consequences for the patient. The two non-study-related adverse events in the automated group during surgery were both associated with surgical maneuvers. There were no significant differences between the manual and automated groups regarding any of these items. A total of four patients (five cases) experienced study equipment malfunctions. No adverse effects were observed in any of these cases.

## Discussion

This study utilized an automated delivery system for TIVA [5] that automatically regulates analgesics, sedatives, and muscle relaxants during TIVA. This approach eliminates subjective influences, such as anesthesiologist preference, and avoids the effects of administering too much or too little anesthetic. We compared the percentage of time that abnormal hypotension occurred in the manually adjusted and automatically controlled groups. The incidence of IOH was markedly high, and all patients recruited in this study received at least one dose of phenylephrine. The percentage of time that the MBP fell below the abnormal hypotension threshold during surgery was significantly lower in the automatically controlled compared to the anesthesiologist-controlled group, indicating statistical non-inferiority. Moreover, the mean values and distribution status of blood pressure in both groups were almost equal, although the automated group was also non-inferior to the manual group regarding the percentage of time that blood pressure rose above the target range during surgery. The automated group was non-inferior to the manual group for the primary end point.

IOH is indeed multifactorial; however, the common practice is to investigate the cause and take effective and rational measures while administering vasopressors to alleviate symptoms. Although an MBP of >65 mmHg has been proposed as an appropriate target for blood pressure control during the perioperative period [1], establishing an optimal target value as in previous research can be challenging due to individual differences (variations in normal MBP) and intraindividual variations (such as intraday fluctuations, fluid intake status, and environmental effects). Under these circumstances, the algorithm adopted in this study—managing blood pressure by addressing abnormal hypotension through measures such as administering a vasopressor when needed—reflects a method that is logical and clinically acceptable. Continuous blood pressure measurement provides substantial data per hour and allows for a shorter evaluation cycle, which generally enhances the accuracy of blood pressure control. However, the number of consecutive blood pressure measurements that resulted in abnormal hypotension and the rate of abnormal hypotension were low in the automated group. This finding suggests that developing an appropriate control algorithm for automated vasopressor administration, based on repetitive measurement data at intervals of several minutes, could be beneficial. These results do not preclude the use of continuous blood pressure monitoring to guide vasopressor administration; rather, it is realistic to utilize continuous and intermittent blood pressure monitoring depending on the clinical situation. To put it to practical use, a safety mechanism is required to remeasure blood pressure values automatically or manually for cases in which they cannot be measured or are unreliable.

Hypertension may occasionally result from an unexpected increase in blood pressure when hypotension is treated with vasopressors. The primary reason for the hypertension observed in this study was the exhibition of high blood pressure at the start of general anesthesia in many cases, which can be attributed to individual patient differences rather than a problem with the control system. Since no hypertension persisted for a prolonged period following the single dose of phenylephrine used in this study, there was no concern regarding the overdosage of vasopressors due to the operation of this system. The algorithm does not allow for medication administration for hypertension; therefore, the research plan required anesthesiologists to administer vasodilators as needed. Considering that no significant difference in the percentage of hypertensive time was observed between the manual and automated groups, using the percentage of hypertensive time to evaluate the effectiveness of the hypotension avoidance system employing this algorithm was deemed insignificant.

Anesthesiologists may not immediately administer a vasopressor in cases of abnormal hypotension while engaged in other tasks. Although this study found no differences between the two groups in total phenylephrine dose, hourly dose, total infusion dose, or hourly infusion dose, the automated group, which received a vasopressor immediately following the onset of abnormal hypotension, recovered more quickly from the abnormal hypotension than the manual group. This quicker response may have contributed to a decreased percentage of abnormal hypotension time. If such an automated administration system for vasopressors is implemented in clinical practice, it is expected to reduce the workload of anesthesiologists in administering vasopressors while enabling rapid and accurate recovery from abnormal hypotension. The causes of hypotension include dehydration due to fasting, insensitive excretion from the surgical site, changes in body distribution due to positional changes, and circulatory depression due to anesthetics. Phenylephrine is also known to increase cardiac output by increasing preload and peripheral vasoconstriction [8], and is effective as an emergency response to decrease circulating blood volume. However, due to the multiple causes of hypotension, reliance on this system alone, which only provides phenylephrine administration, does not guarantee recovery of blood pressure. Anesthesiologists and other medical personnel need to consider the use of additional vasoactive drugs and rapid infusion of fluids in cases where abnormal hypotension persists due to decreased myocardial contractility or preload. Future studies should investigate the adverse effects of increasing the dose of phenylephrine on peripheral blood volume.

**Fig. 8:**
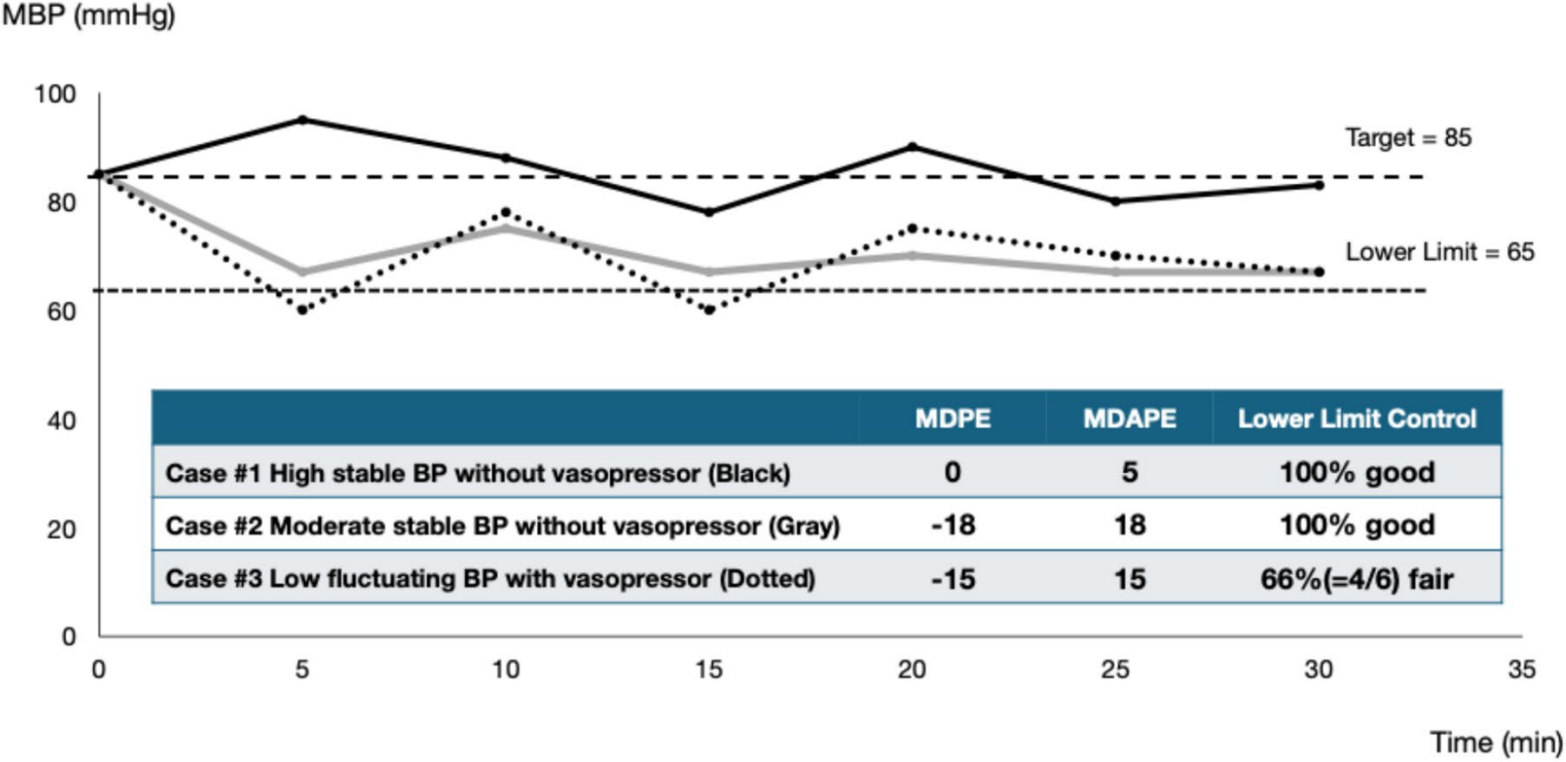
Selection of control accuracy index according to control method. Cases #1 and #2, in which no vasopressor was administered, met the condition of the lower limit control 100% of the time, indicating high control accuracy. However, when comparing them using MDPE and MDAPE, a discrepancy arises in which Case #3 has higher control accuracy than Case #2. *MDPE* median performance error, *MDAPE* median absolute performance error

One limitation of this study is the lack of standardization of infusion administration. Since a standard protocol for infusion administration could not be established and infusion administration was left to the anesthesiologist in charge, the esCCO, which noninvasively predicts stroke volume based on the pulse wave transit time derived from the electrocardiogram and the pulse wave of a pulse oximeter, was recorded as an index of circulatory dynamics. The esCCO correlated well with SV and blood pressure in the phenylephrine-treated condition [9], but there were no apparent differences in cardiac coefficient (esCCI), cardiac output coefficient (esSVI), or infusion rate between the manual and auto-administration groups. However, the influence of infusion therapy was not fully investigated. Therefore, it is necessary to investigate vasopressor administration and appropriate infusion adjustment objectively. Another limitation is that the indicators of control accuracy (median performance error, median absolute performance error, divergence, and wobble) [10] conventionally used in target control cannot adequately evaluate the control state in the lower limit control used in this system (Fig. 8), thereby requiring a new evaluation index. In addition, the current system lacks a function to predict hypotension and administer phenylephrine prophylactically, as vasopressor administration begins only after abnormal hypotension is detected. Integrating a predictive function for prophylactic administration during anticipated hypotension can enhance the ability to prevent abnormal hypotension. While no malfunctions were identified in the device and its safety remains assured, further research is warranted due to the small sample size.

This study confirms that the incidence of hypotension during general anesthesia is notably high and demonstrates that an automated phenylephrine delivery system, which does not rely on invasive arterial pressure measurement, functions effectively. The system's capacity to regulate phenylephrine administration was shown to be comparable to that of anesthesiologists, effectively managing abnormal hypotension while mitigating the risk of hypertension associated with phenylephrine use. This system is anticipated to facilitate the evaluation and study of the clinical benefits of preventing abnormal hypotension during general anesthesia and reduce the workload of anesthesiologists while standardizing blood pressure control in clinical practice.

## Data Availability

Due to the proprietary nature of the technology developed in this study and its relation to intellectual property, the datasets generated and analyzed during the current study are not publicly available. However, summary data and additional information may be provided upon reasonable request, subject to appropriate confidentiality agreements and institutional policies.

## Addendum

The automated delivery system for TIVA is a Software as a Medical Device (SaMD) that controls a group of syringe pumps that deliver three intravenous anesthetics (propofol, remifentanil, and rocuronium) based on information automatically transferred from a biometric monitor. Regarding the control of propofol administration, the propofol effect site concentration was calculated using real-time pharmacokinetic-pharmacodynamic simulation from the start of propofol administration, and a regression curve was obtained in real time from the data set of the propofol effect site concentration and BIS value. The propofol administration rate was changed using target-controlled infusion (TCI) technology to ensure that the effect site concentration would be BIS value = 45 using this regression equation. The remifentanil infusion rate was changed using TCI technology, assuming a concentration at which the rate of decrease in propofol site of action concentration, which becomes BIS=45 due to the increase in effect-site concentration of remifentanil, is small. The rocuronium effect site concentration was calculated using real-time pharmacokinetic-pharmacodynamic simulation from the start of rocuronium administration, and the rocuronium effect site concentration at the point of recovery from complete muscle relaxation to Train-of-four count (TOFC) = 1 was set as the initial target value, and the rocuronium administration rate was changed using TCI technology. This software is currently approved and marketed in Japan as AsisTIVA® but was not approved at the time this study was conducted, and thus it was provided as a specific clinical research study.
